# Depletion of MOB1A/B causes intestinal epithelial degeneration by suppressing Wnt activity and activating BMP/TGF-β signaling

**DOI:** 10.1038/s41419-018-1138-0

**Published:** 2018-10-22

**Authors:** June Sung Bae, Yoon Jeon, Sun Mi Kim, Ji Yun Jang, Mi Kyung Park, In-Hoo Kim, Deog Su Hwang, Dae-Sik Lim, Ho Lee

**Affiliations:** 1 Research Institute, National Cancer Center, Goyang, Gyeonggi 10408 Republic of Korea; 2 Department of Biological Sciences, Seoul National University, Seoul, 08826 Republic of Korea; 3 Graduate School of Cancer Science and Policy, National Cancer Center, Goyang, Gyeonggi 10408 Republic of Korea; 4 Department of Biological Sciences, Biomedical Research Center, Korea Advanced Institute of Science and Technology (KAIST), Daejeon, 34141 Republic of Korea

## Abstract

The Hippo pathway is involved in intestinal epithelial homeostasis with Wnt, BMP, Notch, and EGF signaling. We investigated the relationship between Hippo and other signaling pathways and the role of MOB kinase activator 1A/1B (MOB1A/B) in intestinal homeostasis. Mice with intestinal epithelial cell (IEC)-specific depletion of MOB1A/B showed hyperproliferation in IECs, defects in secretory lineage differentiation and loss of intestinal stem cells and eventually died at 10–12 days after tamoxifen treatment. In MOB1A/B-depleted IECs, expression of Wnt target genes were downregulated but *Bmp2* and *Tgfbr2* were transcriptionally activated with enhanced YAP activity. In in vivo and in vitro experiments with several signaling inhibitors, it has been shown that the BMP inhibitor LDN193189 or TGF-β inhibitor SB431542 had effects on partial restoration of the intestinal degenerative phenotype. Treatment with these inhibitors restored differentiation of secretory lineage cells in MOB1A/B-deficient mice, but not ISC pools in the crypt region. These studies reveal that IEC-specific depletion of MOB1A/B induced overexpression of *Bmp2* and *Tgfbr2* and inhibited Wnt activity, finally leading to loss of ISCs and functional epithelia in the mouse intestine. These results suggest that MOB1A/B has an essential function for intestinal epithelial homeostasis by regulating YAP, Wnt activity, and BMP/TGF-β signaling.

## Introduction

Homeostasis of intestinal epithelial cells is important for maintenance of normal intestinal function. Intestinal stem cells (ISCs) and their highly proliferating progeny, known as transit-amplifying (TA) cells, are responsible for driving epithelial homeostasis and regeneration. Nascent TA cells gradually commit to absorptive or secretory cell lineages while migrating upwards toward the base of the villi. Differentiated cells that exit the crypt region cease to proliferate and then continue migrating upwards along the villi and are ultimately lost via anoikis at the tip of the villi. Functional differentiated cells are renewed every 3–5 days from TA cells except for Paneth cells, which take 3–6 weeks^1^.

Wnt signaling has an essential role in establishing maintenance of stem cells and influencing the regenerative capacity of adult epithelial cells. Following overexpression of the secreted Wnt antagonist Dickkopf 1 (DKK1) in intestinal epithelial cells in mice, epithelial proliferation was decreased and led to loss of crypts^2,3^. Intestinal epithelial cell-specific depletion of TCF4, a transcriptional transactivation partner of β-catenin, induces a complete block of cell proliferation and loss of Lgr5^+^/Olfm4^+^ stem cells^4^. Wnt signaling is stimulated through stabilization of β-catenin in APC-depleted ISCs, and transformed progeny cells are generated more rapidly than in the wild type, eventually resulting in formation of adenomas^5,6^. Wnt activity is also involved in the conversion of ISCs into secretory cell lineages. In the intestine of *Villin-Dkk1* transgenic mice, secretory cell lineages are largely absent^2^. Depletion of Wnt receptor Frizzled-5 induces irregular and random distribution of Paneth cells in crypts and villi^7^. It has been suggested that dysregulation of Wnt activity can impede intestinal homeostasis and trigger the development of cancer or loss of stem cells and secretory cell lineages.

There is negative cross-talk between Wnt and BMP/TGF-β signaling in the intestine. Wnt ligands are primarily expressed in Paneth cells and the mesenchyme surrounding the crypt^8,9^, whereas BMP2 and BMP4 are expressed in mature epithelial cells and the villus mesenchyme, respectively^10,11^. In addition, the BMP inhibitor Noggin is expressed in the crypt region and contributes to create a BMP-low environment surrounding ISCs. Transgenic expression of Noggin induces excessive crypt formation^10,11^. Suppression of BMP signaling, as in the conditional knockout of *Bmpr1a*, induces expansion of stem and progenitor cells with increasing Wnt activity and eventually develops intestinal polyposis^12^. Components of TGF-β signaling are localized in differentiated epithelial cells^13^. In mice, loss of SMAD3 and LTBP-4 lead to development of colorectal cancer^14,15^. BMP/TGF-β signaling can also antagonize Wnt signaling in *Caenorhabditis elegans*^16,17^, mammalian cells^18^ and a human colonic crypt culture model^19^, but additional studies are required to define the in vivo relationship between TGF-β and Wnt signaling in vertebrates. It was recently reported that BMP/TGF-β-induced Smad signaling plays roles in differentiation of diverse epithelial cells and that its inhibition leads to stem cell hyperplasia in human and mice^20^. Finally, it has been suggested that inhibition of Wnt signaling by BMP/TGF-β signaling may regulate differentiation and homeostasis of a broad spectrum of epithelial cell types in addition to intestinal epithelial cells (IECs).

The Hippo signaling pathway plays a crucial role in cell proliferation, apoptosis, differentiation, and development. Large tumor suppressor 1 and 2 (LATS1/2) kinase is activated by binding MOB1A/B^21^ and then phosphorylate Yes-associated protein 1 (YAP). The transcriptional activity of phosphorylated YAP is inhibited by cytoplasmic sequestration with the 14-3-3 protein and by SCF^beta-TrCP^ E3 ubiquitin ligase-dependent degradation^22^. The exact function of Hippo signaling remains controversial in intestinal homeostasis and cancer. It has been reported that expansion of undifferentiated progenitor cells and dysplasia with Wnt signaling activation are induced in the intestines of *Yap* transgenic mice and IEC-specific MST1/2 knockout mice^23,24^. These results support the idea that YAP functions as an oncogenic transcriptional coactivator. Moreover, it has been known that YAP promotes the proliferation of stem and progenitor cells through repression of *Yap* using intestine-specific gene transfer methods^25^. Paradoxically, Barry et al.^26^ reported that overexpression of YAP in IECs repressed Wnt signaling activity and induced loss of proliferating crypts and ISCs. Therefore, further studies are required to investigate the roles of Hippo signaling and YAP transcriptional activity in intestinal homeostasis using additional model systems.

A number of studies have reported Hippo signaling and YAP cross-talk with other biological signaling pathways, including Wnt, BMP, TGF-β, Notch, and EGF^27^. However, few studies have focused on whether and how Hippo signaling controls other biological signaling or vice versa in intestinal epithelial homeostasis and regeneration. We found that activation of YAP and TAZ by MOB1A/B depletion suppressed Wnt activity, enhanced BMP/TGF-β signaling and led to collapse of *in vivo* intestinal epithelial homeostasis in the mouse intestine. Inhibition of BMP/TGF-β in the intestine using chemical drugs partially restored Wnt activity and secretory cell lineages. These results reveal the essential roles of Hippo signaling in ISCs and IECs regarding intestinal homeostasis and regeneration.

## Results

### MOB1A/B is essential for homeostasis in intestinal epithelial cells

**Fig. 1:**
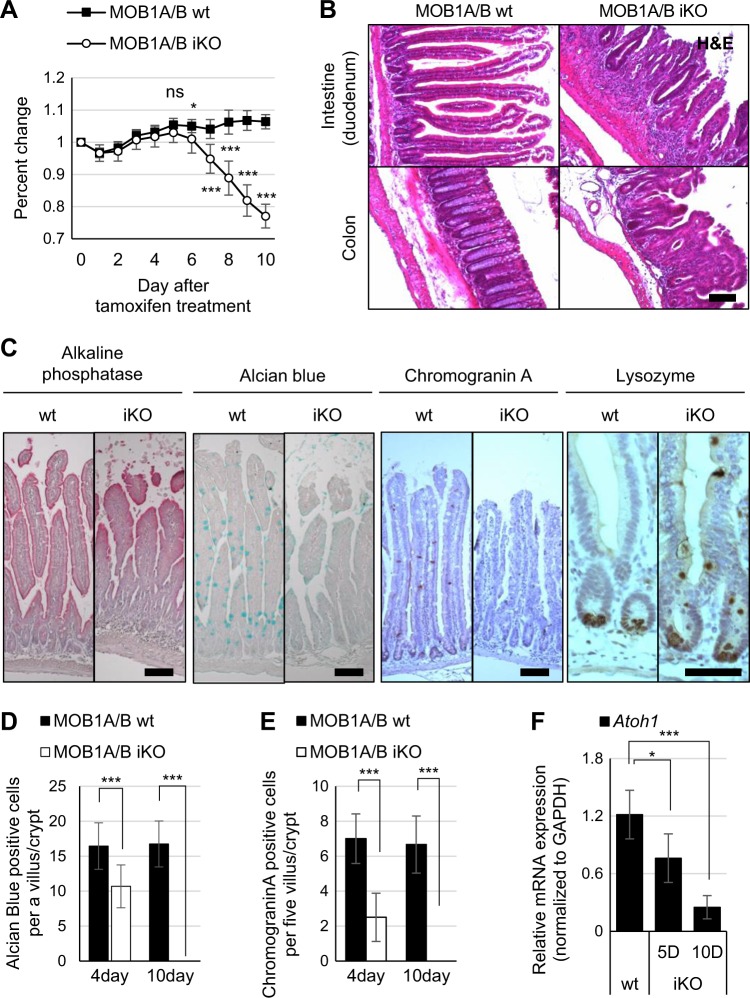
MOB1A/B is essential for homeostasis of the intestinal epithelium. **a** Change in body weight of wild type (*n* = 10) and MOB1A/B iKO mice (*n* = 17) after tamoxifen treatment. **b** Representative hematoxylin and eosin (H&E) staining in control and MOB1A/B-depleted intestines sampled 10 days after tamoxifen treatment (80 mg/kg per day, during 2 days). *Scale bars*, 100 μm. **c** Staining for secretory cell lineage in the intestine seven days after tamoxifen treatment. From left to right; alkaline phosphatase, Alcian blue staining, IHC staining of intestines with anti-Chromogranin A and anti-Lysozyme antibody. *Scale bars*, 50 μm. **d, e** The number of Alcian blue- and Chromogranin A-positive cells in three individual control and MOB1A/B-depleted mice. **f** Relative mRNA expression levels of *Atoh1* in isolated IECs of control and MOB1A/B iKO mice (*n* = 3). Data are presented as the mean ± SEM. ^*^P < 0.05, ^***^P < 0.001

To investigate the roles of MOB1A/B in IECs, a *Mob1a/b* conditional knockout mouse was generated and bred with a *Villin-cre* transgenic mouse (Supplementary Figure S1). However, no viable pups were obtained, indicating that MOB1A/B is essential for normal development of IECs during embryogenesis (Table S1). To avoid these developmental defects during embryogenesis, *Mob1a*^f/f^;*Mob1b*^f/f^;*Villin-creER*^T2^ mice (hereafter designated as MOB1A/B iKO mice) were generated and analyzed in further studies. MOB1A/B iKO mice were born in a Mendelian ratio and were healthy and fertile. Tamoxifen was intraperitoneally injected 8 weeks after birth to induce deletion of the *Mob1a/b* gene in mouse IECs. While there was little change in the body weight of wild-type mice, weight of MOB1A/B iKO mice was continually decreased from 6 days after treatment. Mutant mice were near death with hematochezia and finally died at 10–12 days after treatment (Fig. 1a). In MOB1A/B iKO mice, the crypts of the small and large intestines were drastically reduced in size and number and the intestinal epithelium was structurally collapsed (Fig. 1b). Although the abundance of absorptive enterocytes was mostly preserved, the whole structure of the intestinal villus was disorganized, with a marked decrease in goblet cells and enteroendocrine cells with randomly distributed Paneth cells in the crypt and villi (Fig. 1c). At 10 days after tamoxifen treatment, goblet cells and enteroendocrine cells completely disappeared in whole IECs (Fig. 1d, e). Consistent with these observations, *Atoh1* which is expressed in secretory epithelial cell types and progenitors was downregulated in IECs isolated from MOB1A/B iKO mice (Fig. 1f). These results suggest that MOB1A/B is required for normal function of the intestine and colon by regulating the homeostasis of secretory cell lineages and maintenance of the crypt structure.

### Loss of MOB1A/B leads to YAP activation and hyperplasia in the crypt and villus

**Fig. 2:**
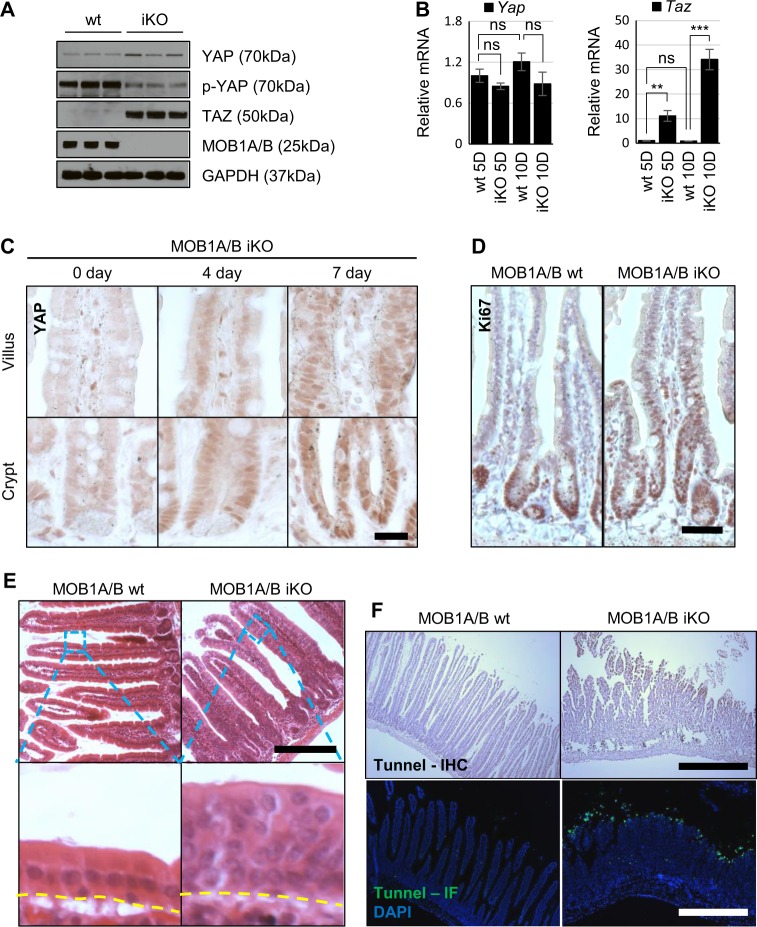
Loss of MOB1A/B leads to YAP activation and hyperplasia in the crypt and villus. **a** Western blot analysis of IECs isolated seven days after tamoxifen treatment in three individual control and MOB1A/B iKO mice. **b** Relative expression levels of *Yap* and *Taz* mRNA in 5 or 10 days after tamoxifen treatment in three individual control and MOB1A/B iKO mice. **c** Representative IHC staining of intestines with anti-YAP antibody at the indicated times after tamoxifen treatment. *Scale bars*, 20 μm. **d** Representative IHC staining of intestines with anti-Ki67 antibody at 4 days after tamoxifen treatment. *Scale bars*, 50 μm. **e, f** H&E staining (**e**) and TUNEL staining (**f**) of control and MOB1A/B-depleted intestines seven days after tamoxifen treatment. The upper regions of the yellow dotted lines indicate IECs. *Scale bars*, 200 μm (**e**) and 500 μm (**f**), respectively. Data are presented as the mean ± SEM. ^**^P < 0.01, ^***^P < 0.001

MOB1A/B with MST1/2, SAV1 and LATS1/2 are Hippo core components in mammals^28^. To verify whether depletion of MOB1A/B induces YAP or TAZ activation, YAP and TAZ protein levels were measured in IECs isolated from MOB1A/B iKO mice. The loss of MOB1A/B resulted in a slight increase in total YAP protein despite a dramatic decrease in YAP Ser127 phosphorylation and a significant increase in the total amount of TAZ protein (Fig. 2a). Interestingly, transcription of the *Taz* gene was dramatically upregulated in MOB1A/B-deficient IECs, whereas there was little change in transcription of the *Yap* gene (Fig. 2b). Transcriptional upregulation of *Taz* has also been reported in SAV1-deficient IECs and was completely reversed in *Sav1;Yap* double-mutant crypts^29,30^. These results suggest that TAZ activity can be enhanced by upregulation of its own protein levels, which may be increased due to other transcriptional factor(s), such as YAP, in MOB1A/B-deficient IECs. YAP activity can be posttranscriptionally regulated via phosphorylation by MOB1A/B-LATS kinase.

YAP-expressing and Ki67-stained cells were localized in the crypt region of the normal intestine (Fig. 2c, d). However, YAP was localized prominently in the nucleus, and YAP-expressing and Ki67-stained cells expanded toward the tip in the villi of MOB1A/B-depleted IECs (Fig. 2c, d). At 7 days after tamoxifen treatment, hyperplasia was observed in IECs (Fig. 2e). Given that epithelial hyperplasia has been reported to occur in MST1/2-deficient IECs and the intestines of *Yap* transgenic mice^23,24^, it is likely that MOB1A/B-depleted IECs develop hyperplasia due to increased YAP activity. We also investigated whether the hyperactive YAP has an anti-apoptotic function in IECs. However, noticeable increased cell death at the tip of the villi, or anoikis, was observed 10 days after tamoxifen treatment (Fig. 2f). These data suggest that MOB1A/B depletion induces activation of YAP in the crypt and villus, followed by hyperplasia and increased anoikis in the intestine.

### Depletion of MOB1A/B causes downregulation of stem cell niche factors leading to degeneration of ISCs

**Fig. 3:**
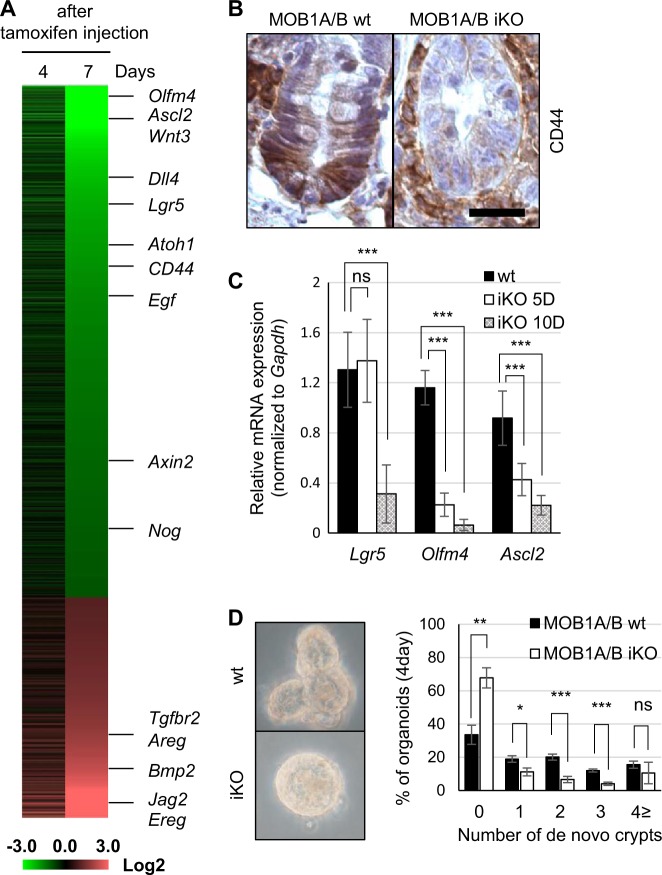
Depletion of MOB1A/B induces changes in stem niche factors leading to degeneration of ISCs/crypt. **a** Heatmap of IECs isolated from tamoxifen-treated mice (*n* = 2) for the indicated periods showing 13,700 downregulated genes and 5912 upregulated genes in rank order of fold change value at 7 days. Labeled genes are examples known to be involved in Wnt, Notch, EGF, TGF-β, and BMP signaling and ISCs markers. **b** Representative IHC staining with anti-CD44 antibody at the 7 days after tamoxifen treatment in three individual control and MOB1A/B-depleted mice. *Scale bars*, 20 μm. **c** Relative mRNA expression levels of ISCs marker genes (*Lgr5, Olfm4*, and *Ascl2*) in isolated IECs of control and MOB1A/B iKO mice (*n* = 3). **d** Representative bright-field images of intestinal organoids and the percentage of organoids showing 0–3 or ≥4 de novo crypt formation. MOB1A/B-depleted ISCs were isolated from control or tamoxifen-treated MOB1A/B iKO mice and analysis of crypt formation was performed 4 days after ISCs isolation (*n* = 3, *n* represents the number of separate cultures). Data are presented as the mean ± SEM. ^*^P < 0.05, ^**^P < 0.01, ^***^P < 0.001

To further investigate the cause of defects in IECs homeostasis and acute death of MOB1A/B iKO mice, microarray analysis with isolated IECs was performed (Fig. 3a). Expression of intestinal stem niche factors, *Wnt3* (Wnt Family Member 3), *Dll4* (Delta Like Canonical Notch Ligand 4), *Egf* (Epidermal Growth Factor), and *Nog* (Noggin), were suppressed in MOB1A/B iKO IECs (Fig. 3a). This analysis also revealed that expression of Wnt target genes (*Axin2* and *Cd44*) was dramatically decreased in the IECs of MOB1A/B iKO mice, implying that loss of MOB1A/B in IECs induces suppression of Wnt activity. Reduced expression of CD44 was confirmed in histological analysis of IECs from MOB1A/B iKO mice (Fig. 3b). Given that expression of ISCs markers (e.g., *Lgr5, Olfm4,* and *Ascl2*) is dependent on Wnt activity^31^, expression of these markers was evaluated in MOB1A/B iKO IECs. Using microarray analysis and quantitative RT-PCR, it was found that *Lgr5, Olfm4*, and *Ascl2* were downregulated in IECs isolated from MOB1A/B iKO mice (Fig. 3a–c).

To investigate whether MOB1A/B depletion in IECs influenced not only downregulation of ISCs markers but also crypt formation, we performed in vitro organoid cultures using IECs isolated from wild type and MOB1A/B iKO mice. Normal crypt budding was observed in the wild-type organoid, but reduced crypt budding was observed in MOB1A/B iKO organoids (Fig. 3d). These results suggest that MOB1A/B has essential functions for maintaining Wnt activity and the organization of crypt structure. Given that Wnt signaling has been shown to play roles in the fate decisions of secretory lineages and Paneth cell differentiation^2,7,32^, MOB1A/B-Wnt or Hippo-Wnt activity may have regulatory functions in differentiation of ISCs and/or progenitor cells and organization of crypt structures.

### Wnt activity is suppressed in MOB1A/B-deficient ISCs

**Fig. 4:**
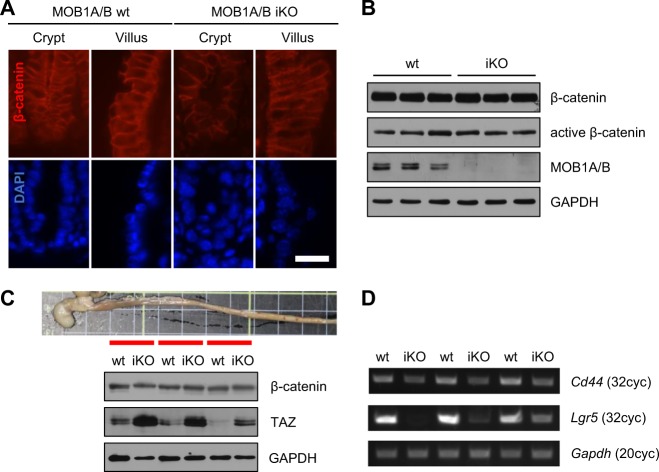
MOB1A/B depletion reduces β-catenin activity. **a** Representative IHC staining with anti-β-catenin antibodies in control and MOB1A/B-depleted intestines sampled 7 days after tamoxifen treatment. *Scale bars*, 20 μm. **b** Western blot analysis of IECs isolated at 7 days after tamoxifen treatment in three individual control and MOB1A/B iKO mice. **c, d** Western blot analysis (**c**) and semiquantitative PCR analysis (**d**) of IECs isolated 2 cm long segments duodenum—jejunum region at 7 days after tamoxifen treatment in three individual control and MOB1A/B iKO mice

Cytoplasmic YAP and TAZ are components of the β-catenin destruction complex^33,34^ and sequester β-catenin in the cytoplasm^35^. The intensity of β-catenin immunofluorescent staining decreased slightly in the crypt region of MOB1A/B iKO mice (Fig. 4a). However, little difference was detected in the levels of total β-catenin or active β-catenin (dephospho-Ser45) between control and mutant IECs (Fig. 4b). The β-catenin protein did not decrease in the 2 cm long segments of the proximal intestinal region, despite an increase in the level of the TAZ protein (Fig. 4c). Although the high expression level of YAP and TAZ in MOB1A/B-depleted IECs were accompanied by little or no change in β-catenin stability, Wnt target genes and ISC markers decreased more markedly in the microarray analysis and quantitative RT-PCR, indicating suppression of β-catenin transcriptional activity in mutant IECs.

Because Wnt signaling has been known to be crucial for the maintenance of adult crypt proliferation^2,3^, we assumed that if MOB1A/B was depleted ISCs and TA cells would lose their stem cell/progenitor characteristics and disappear due to cell death. To verify this hypothesis, *Mob1a*^f/f^;*Mob1b*^f/f^;*Villin-creER*^*T2*^;*R26R-LacZ* mice were generated for a lineage tracing assay, and the depletion of MOB1A/B was induced in partial subset of IECs by treatment with low-dose tamoxifen (40 mg/kg, once). This dosage was at the threshold of the nonlethal tamoxifen dose in MOB1A/B iKO mice. A marked decrease in MOB1A/B-depleted LacZ-positive cells was observed 4 weeks after the low-dose tamoxifen treatment, compared to the control (Supplementary Figure S2). Taken together, these results indicate that MOB1A/B-depleted, Wnt activity-suppressed ISCs were negatively selected.

### Inhibition of TGF-β or BMP signaling partially restores degeneration phenotypes by MOB1A/B depletion

Few crypt structures were observed at 10 days after high-dose tamoxifen treatment in MOB1A/B iKO mice (Fig. 1c) and MOB1A/B depletion induced marked downregulation of Wnt target genes, including ISC markers (Fig. 3a–c). However, MOB1A/B depletion did not suppress the stability and phosphorylation status of β-catenin in spite of activation of YAP and TAZ, which are components of the β-catenin destruction complex. Therefore, it is assumed that another factor in the crypt region contributed to suppression of β-catenin transcriptional activity followed by depletion of ISCs population.

**Fig. 5:**
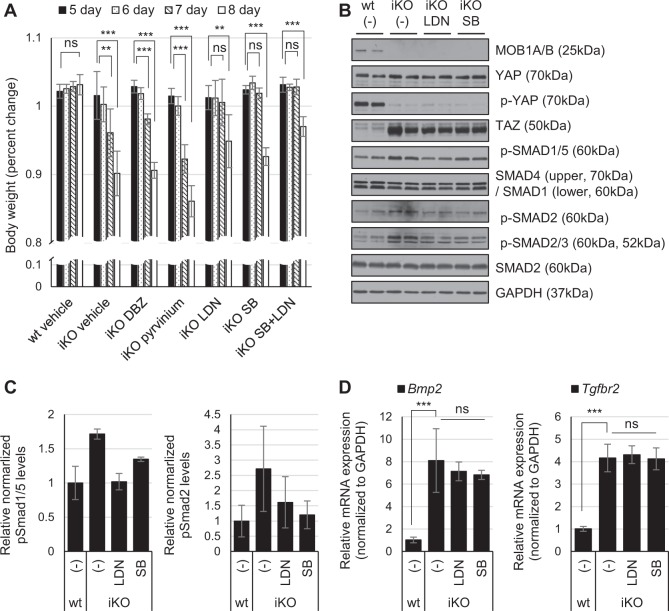
Inhibition of TGF-β or BMP signaling delays the decrease in body weight of MOB1A/B-depleted mice. **a** Percent changes in body weight of vehicle-treated wild type and vehicle- or inhibitor-treated MOB1A/B iKO mice after tamoxifen treatment. Vehicle-treated wild-type mice, *n* = 6; vehicle-treated MOB1A/B iKO mice, *n* = 8; dibenzazepine (DBZ)-treated MOB1A/B iKO mice, *n* = 6; pyrvinium-treated MOB1A/B iKO mice, *n* = 6; LDN-treated MOB1A/B iKO mice, *n* = 7; SB-treated MOB1A/B iKO mice, *n* = 6; combination treatment with LDN and SB in MOB1A/B iKO mice, *n* = 6. **b** Western blot analysis of IECs isolated 6 days after treatment with tamoxifen and indicated inhibitors in control and MOB1A/B iKO mice. **c** Quantification of protein levels for pSmad1/5 and pSmad2 from (**b**). **d** Quantitative results showing expression levels of *Bmp2* and *Tgfbr2* mRNA isolated from IECs in (**b**). Data are presented as the mean ± SEM. ^**^P < 0.01, ^***^P < 0.001

Because YAP and TAZ function as co-transcriptional factors, we focused on transcriptionally enhanced genes in microarray analysis with MOB1A/B iKO IECs. The abundance of *Jag2*, a ligand of Notch signaling, *Bmp2*, a ligand of BMP signaling, and *Tgfbr2*, a receptor of TGF-β signaling, were significantly upregulated by MOB1A/B depletion (Fig. 3a–d). Several groups have reported a negative regulatory mechanism between Notch and Wnt^36^ or BMP/TGF-β and Wnt^12,16–19^. To investigate whether Notch or BMP/TGF-β signaling are involved in suppression of Wnt activity or depletion of ISCs populations, γ-secretase inhibitor dibenzazepine (DBZ), BMP receptor inhibitor LDN193189 (LDN), and TGF-β receptor inhibitor SB431542 (SB) were used to treat MOB1A/B iKO mice. In addition, we assumed that the Wnt inhibitor (Pyrvnium) promotes intestinal defects of MOB1A/B knockout mice. However, the Pyrvinium had no effect on decrease in the body weight of MOB1A/B knockout mice. Therefore, MOB1A/B depletion and Pyrvinium may not be synergetic in the suppression of Wnt activity or MOB1A/B depletion would have already completely inhibited the Wnt activity (Fig. 5a).

Whereas DBZ had no effect on recovery of body weight in MOB1A/B iKO mice, treatment of LDN and SB had partial effects (Fig. 5a). We were not observed changes in body weight in LDN- or SB-treated MOB1A/B wt mice (Supplementary Figure S3a). Phosphorylated SMAD proteins, indicative of activation of BMP/TGF-β signaling, were decreased in IECs isolated from LDN- or SB-treated MOB1A/B iKO mice (Fig. 5a–c). No significant changes were observed in LDN- or SB-treated MOB1A/B wt mice except for downregulation of phospho-SMADs (Supplementary Figure S3). Notably, inhibiting BMP/TGF-β signaling caused little changes in the expression levels or localization of YAP and TAZ, YAP Ser127 phosphorylation, or expression of *Bmp2* and *Tgfbr2* in MOB1A/B iKO mice (Fig. 5b–d and Supplementary Figure S4). We also observed the upregulation of *Bmp2* and *Tgfbr2* by MOB1A/B knockdown in the Caco-2 colon cancer cell line. These abundance of *Bmp2* and *Tgfbr2* was dependent on YAP, and TAZ activities (Supplementary Figure S5). Taken together, these results suggest that increased expression of *Bmp2* and *Tgfbr2* in MOB1A/B-depleted IECs activates BMP/TGF-β signaling and the treatment of LDN or SB specifically inhibit the *Bmp2*/*Tgfbr2* mediated phosphorylation of SMADs in vivo.

**Fig. 6:**
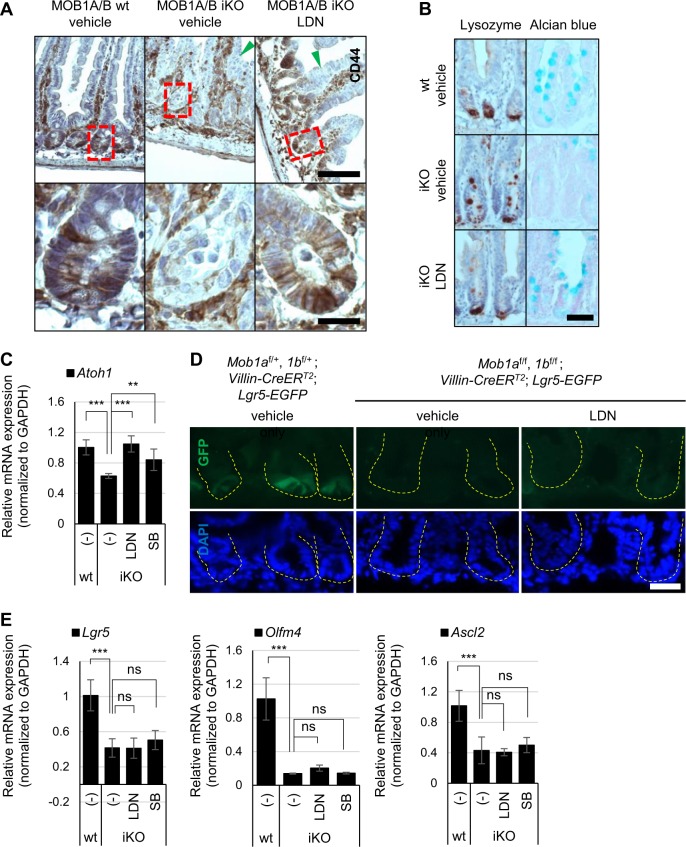
Inhibition of TGF-β or BMP signaling restores defects in secretory lineage differentiation in MOB1A/B-depleted IECs. **a** Representative IHC staining with anti-CD44 antibodies in three individual control and MOB1A/B-depleted intestines sampled 7 days after treatment with tamoxifen and/or indicated inhibitors. The lower images are an enlarged view of red-dotted box in the upper images. *Scale bars*, 100 μm (upper) and 20 μm (lower). **b** Representative IHC staining with anti-CD44 antibodies and Alcian blue staining in three individual control and MOB1A/B-depleted intestines sampled 7 days after treatment with tamoxifen and/or indicated inhibitors. *Scale bars*, 20 μm. **c** Quantitative results showing expression levels of *Atoh1* mRNA from IECs isolated from LDN- or SB-treated control and MOB1A/B iKO mice (*n* = 3). **d** Representative immunofluorescence image of intestines of indicated mice (*n* = 3). Dashed lines depict the crypt structure. *Scale bars*, 20 μm. **e** Quantitative results showing expression levels of *Lgr5*, *Olfm4*, and *Ascl2* mRNA from IECs isolated from LDN- or SB-treated control and MOB1A/B iKO mice (*n* = 3). Data are presented as the mean ± SEM. ^**^P < 0.01, ^***^P < 0.001

Because the reduction of body weight was delayed by treating LDN or SB, we hypothesized that the inhibition of BMP/TGF-β signaling can recover the intestinal defects caused by depleting MOB1A/B. In histological analysis, LDN treatment mostly rescued Paneth and goblet cells and reversed repression of CD44 in the crypt epithelial region in MOB1A/B-depleted intestine (Fig. 6a, b). In in vitro organoid culture, LDN or SB treatment increased crypt formation of MOB1A/B iKO organoids (Supplementary Figure S6a). mRNA levels of *Atoh1*, which are indicative of secretory cell lineages and progenitors, and *Muc2*, which are indicative of goblet cell marker, were increased in IECs of LDN- or SB-treated MOB1A/B iKO mice (Fig. 6c and Supplementary Figure S6b). These results suggest that inhibition of BMP/TGF-β signaling restores defects in differentiation into secretory cell lineages and Wnt activity caused by MOB1A/B depletion.

To investigate whether ISCs are recovered by BMP/TGF-β signaling inhibition (LDN treatment), *Mob1a*^f/f^;*Mob1b*^f/f^;*Villin-creER*^*T2*^;*Lgr5-EGFP* mice were generated. We found that Lgr5-positive stem cells vanished after tamoxifen treatment (80 mg/kg per day, during 2 days) in this mouse model (Fig. 6d). Depletion of Lgr5-positive stem cells was not restored by BMP/TGF-β signaling inhibition (LDN treatment) (Fig. 6d). mRNA levels of *Lgr5*, *Olfm4*, and *Ascl2*, which are indicative of ISCs, were also not upregulated in IECs of LDN- or SB-treated MOB1A/B iKO mice (Fig. 6e). These results suggest that the delayed decreased body weight in LDN- or SB-treated MOB1A/B iKO mice was due to comparatively improved maintenance of functional secretory cell lineages and progenitors.

Taken together, we suggest that the Hippo core components MOB1A/B or LATS/MOB1 have an essential function in maintaining intestinal epithelial homeostasis by regulating Wnt activity and BMP/TGF-β signaling.

## Discussion

It has been recently reported that loss of LATS1/2 or MOB1A/B forces hepatoblasts or hepatocytes to commit to biliary epithelial cell lineage and elevated TGF-β production with YAP and TAZ activation in the liver. Treatment with a TGF-β receptor inhibitor or TGF-β receptor-null mutation in the liver suppressed these abnormalities^37,38^. YAP/TAZ-TEAD complex also binds to the *Tgfb2* locus and directly regulates the transcription of *Tgfb2*^38^. Similar to TGF-β signaling, BMP signaling is also associated with the Hippo pathway. *Bmp4* is transcriptionally activated by TAZ/TEAD, which promotes SMAD1/5/8-mediated signaling in breast epithelial cell lines^39^. YAP also enhances SMAD1-dependent transcription in cell culture systems^40^. Our studies identified that IECs-specific MOB1A/B depletion enhances expression of *Bmp2* and *Tgfbr2* in IECs. We assumed that excessive BMP2 and TGFβR2 expression, which can overcome activity of the antagonist Noggin, could be a prerequisite for the loss of secretory cell lineages and ISCs as well as suppressing Wnt activity in MOB1A/B-depleted IECs.

EGF is a potent stimulator of cell proliferation in epithelial and nonepithelial cell types present throughout the gastrointestinal tract^41^ and is a crucial component of intestinal organoid culture^42^. The EGF family of receptor tyrosine kinases (ErbBs) also plays an essential role in proliferation of intestinal stem/progenitor cells^43^. Activation of ErbB signaling caused by loss of Lrig1 results in rapid ISCs expansion^44^. We observed that loss of MOB1A/B in IECs reduced the expression of intestinal stem niche factors, which include EGF. However, transcription of other EGFR ligands, *Areg* (Amphiregulin) and *Ereg* (Epiregulin), were markedly upregulated in MOB1A/B iKO IECs (Fig. 3a). Given previous results showing that *Areg* and *Ereg* transcription can be induced by YAP or TAZ activity^45,46^, these results support that EGF signaling is interactive with Hippo signaling. After irradiation, nuclear localization and increased transcriptional activity of YAP were observed during intestinal regeneration. In this process, one of the YAP-dependent upregulated genes was *Ereg*. Exogenous Ereg rescued YAP loss phenotype in organoid formation assay and elevated stromal *Ereg* was observed around survived YAP-depleted crypts after irradiation^46^. Our results that treatment with a BMP/TGF-β inhibitor did not restore the dysregulation of EGFR family ligands (Supplementary Figure S6c) and degenerative phenotype of ISCs (Fig. 6d, e) support that additional signaling, such as EGF function, are involved in the identity and maintenance of ISCs in addition to BMP/TGF-β and/or Wnt signaling. Restoring crypt formation in LDN- or SB-treated MOB1A/B iKO organoids can be explained by sufficient additives, such as EGF in the culture media (Supplementary Figure S6a). Further studies are required to verify whether or how EGF signaling functions in ISCs maintenance and how Hippo signaling interacts with EGF signaling in IECs.

It was confirmed that depletion of MOB1A/B induced upregulation of *Bmp2* and *Tgfbr2* and activated BMP- and TGF-β-induced SMAD signaling in mouse IECs, as assessed by increased phosphorylation of SMAD2/3 and SMAD1/5 (Fig. 5b). To investigate whether Wnt suppression is mediated by activation of BMP/TGF-β signaling in MOB1A/B iKO mice, the BMP/TGF-β signaling inhibitors LDN and SB were evaluated. It has been known that LDN is a selective inhibitor of BMP-activated ALK2/3/6, in which activation primarily phosphorylates SMAD1, 5, and 8^47^. SB is a potent and selective inhibitor of TGF-β-activated ALK4/5/7, in which activation largely phosphorylates SMAD2 and 3^48^. However, the specificity of SMAD phosphorylation by BMP/TGF-β is less stringent^49–51^ and the selectivity of LDN or SB is not fixed. For example, LDN also inhibits ALK4 and 5, even if the IC_50_ is higher than ALK2 in vitro^52^. Treatment of LDN inhibits BMP2-induced phosphorylated SMAD2/3, and SB inhibits TGF-β1-induced phosphorylated SMAD1/5 in a myoblast cell line^53^. In this study, LDN repressed phosphorylation of SMAD2 as well as SMAD1/5 and SB inhibited phosphorylation of SMAD1/5 as well as SMAD2 (Fig. 5b). LDN treatment reversed repression of CD44 in the crypt even if epithelial hyperplasia along the villus axis by oncogenic activity of YAP and TAZ was still observed (Fig. 6a). We suggest that LDN or SB treatment in MOB1A/B iKO mice can restore Wnt activity in IECs via suppressing activation of both BMP and TGF-β signaling.

In summary, we identified a role of the Hippo core component MOB1A/B that is essential to maintain stem cell populations and generate secretory cell lineages in the mouse intestine. Our results suggest that MOB1A/B suppresses YAP/TAZ activity as well as BMP/TGF-β signaling, in which activation results in inhibition of Wnt activity in the crypt region and loss of intestinal epithelial homeostasis. These findings will be important to shed light on novel Wnt suppression mechanisms by Hippo signaling in IECs and to determine which signals associate and interact together to influence intestinal epithelial homeostasis.

## Materials and methods

### Generation of *Mob1a* and *Mob1b* conditional knockout mouse

Targeting strategies are described in Supplementary Figure S1. Generation of targeted ES cell clones and germline transmission of the *Mob1a*^*puro*^ and *Mob1b*^*puro*^ alleles were performed according to the methods as previously described^54,55^. All mice strains were backcrossed more than six generation to C57BL/6J. This study was reviewed and approved by the Institutional Animal Care and Use Committee (IACUC) of the National Cancer Center Research Institute. PCR primers for genotyping are described in Supplementary Figure S1.

### Histology, immunohistochemistry, and immunofluorescence analysis in paraffin section

Intestines and colons of 8–9-weeks-old mice were isolated and fixed at 4 °C for overnight with fresh 4% paraformaldehyde (PFA) in PBS. The intestinal tissues were then embedded in paraffin. Five micrometer paraffin sections were prepared using a microtome and stained with hematoxylin and eosin. For immunohistochemical staining, the sections were deparaffinized and rehydrated using the standard protocol. Antigen retrieval was performed in 10 mM trisodium citrate, pH 6.0/0.05% tween 20 solution by boiling for 10 min with microwave. The tissue sections were incubated with blocking solution (10% goat serum, 1% BSA/TBS) for 1 h at room temperature and reacted with primary antibodies (Table S2) at 4 °C for overnight and corresponding biotinylated secondary antibodies diluted 1:500 in 1% BSA/TBS at room temperature for 1 h. The slides were incubated in 0.3% H_2_O_2_ in TBS for 15 min to block endogenous peroxidase. ABC avidin-biotin-DAB detection kit (Vector Labs, Burlingame, CA, USA) was then used for detection and visualization of staining according to the supplied protocol. Finally, slides were counterstained with hematoxylin and dehydrated for coverslip mounting. For immunofluorescent staining, sections were incubated in Alexa Fluor^®^ 568 conjugated secondary antibody (Thermo Fisher Scientific, Waltham, MA, USA) at room temperature for 40 mins and then mounted with VECTASHIELD^®^ mounting medium with DAPI (Vector Labs). Staining of alkaline phosphatase activity was performed with the Vector^®^ RED substrate kit and counterstained with hematoxylin. For Alcian blue staining, the sections were immersed in Alcian blue solution (Sigma, St. Louis, MO, USA) for 30 min at room temperature and counterstained with the Nuclear fast red solution (Vector Labs). Images were obtained using Observer.Z1 or Imager.M1 (Zeiss, Oberkochen, Germany).

### Frozen section for immunofluorescence microscopy

Tube-shaped intestine tissues were cut lengthwise and opened such that the lumen of the intestine faces upon 3 mm paper. Tissues fixed on paper were incubated in 2% PFA at 4 °C for 1 h. The tissues were rinsed with PBS 3 times (10 min each) and incubated in sucrose/O.C.T. solution (15% sucrose, 50% Tissue-Tek^®^ O.C.T. (Sakura Finetek, Tokyo, Japan) in PBS) at 4 °C for 1 h. Afterward, the tissues were frozen in O.C.T. compound for cryosectioning of 10 µm thickness. The slides were stained with standard immunofluorescent staining protocol.

### Harvesting intestinal epithelial cells and immunoblot analysis

Proximal intestines (6 cm) were harvested and any membrane, blood vessels, and fat from the exterior of the intestines removed. Tissues were cut into 2 mm segments and incubated in 2 mM EDTA/PBS solution for 1 h at 4 °C on a shaker. After removal of EDTA medium, epithelial cells were released by repeated vigorous shaking in cold PBS and harvested except for mucosal- and submucosal-layer. Isolated epithelial cells were lysed with RIPA buffer (GenDepot, Barker, TX, USA) containing Xpert proteinase inhibitor cocktail and phosphatase inhibitors (GenDepot). The protein concentration in each lysate was quantified with a protein assay dye reagent (Bio-Rad, Hercules, CA, USA). Blots were incubated with primary antibodies in 0.05% Tween 20/TBS (TBST)-based solution (Table S3) at 4 °C for overnight on a shaker and corresponding HRP-conjugated secondary antibodies (GenDepot) at room temperature for 40 min on shaker. Immunoblot analysis was performed with the standard protocol.

### Microarray analysis

Microarray analysis was performed in eBiogen Inc. (Seoul, Republic of Korea). For each RNA, synthesis of target cRNA probes and hybridization were performed using Agilent’s LowInput QuickAmp Labeling Kit (Agilent Technologies, Santa Clara, CA, USA) according to the manufacturer’s instructions. The fragmented cRNA was hybridized onto assembled Agilent Mouse (V2) Gene Expression 4 × 44 K GeneChips (Agilent Technologies). The hybridization images were analyzed by Agilent DNA microarray Scanner and the data quantification was performed using Agilent Feature Extraction software 10.7. The number of Transcript profiling is GSE103338. All data normalization and selection of fold-change genes were performed using GeneSpringGX 7.3.1. (Agilent Technologies). Functional annotation of genes was performed according to Gene OntologyTM Consortium (http://www.geneontology.org/index.shtml) by GeneSpringGX 7.3.1.

### The organoid culture of IECs

For isolation of crypt epithelial cells, EDTA treated intestinal tissue fragments were vigorous suspension by using a 1 ml blunt tip with cold PBS. The supernatant was the villous fraction and discarded (repeat twice). After a further vigorous suspension of tissue sediment, the supernatant was enriched for crypts epithelial cells. This fraction was passed through a 70 μm cell strainer to remove residual villous material. Isolated crypts were centrifuged at 150–200 g for 3 min to separate crypts from single cells. For organoid formation, isolated crypts were cultured using IntestiCultTM Organoid growth Medium (STEMCELL Technologies, Vancouver, BC, Canada), growth factor reduced Matrigel (Corning, Bedford, MA, USA) and DMEM/F12 (Welgene, Gyeongsan, Republic of Korea) according to manufacturer’s instruction.

### Cell culture and transfection

Caco-2 colon cancer cell lines were obtained from the Korean Cell Line Bank and cultured in Minimum Essential Medium supplemented with 10% fetal bovine serum (Sigma). pLKO.1-puro and pLKO.1-blast lentiviral vectors for shRNAs targeting human *Mob1a* and *Mob1b* were gifts from Bob Weinberg and Keith Mostov (Addgene, Cambridge, MA, USA), respectively^56,57^. shRNA target sequences used in this study are as followed. shMob1a#1 (CAGCTTGATGATGAAACTCTT), shMob1a#2 (GGTTAACCTGTAGCTTATAAA), shMob1b#1 (TGATTATGTGAAACCATATTC), and shMob1b#2 (CAATCAGATCAACATGCTTTA). Annealed oligomers were cloned into pLKO.1 vector and these plasmids were transfected using JetPEI^®^ transfection reagent (Polyplus, Illkirch, France) in 293FT cell lines with pLP1, pLP2, and pLP/VSVG plasmids (Invitrogen). Viral supernatant transduced the Caco-2 cell lines and 2 μg/ml puromycin or 20 μg/ml blasticidin was added in the media for selection. For siRNA mediated knockdown, human siRNA were purchased from Santa Cruz Biotechnology. siRNA sequences used in this study are as followed. siControl (sc-37007), siYAP (sc-38637), and siTAZ (sc-38568). siRNAs were transfected using Lipofectamine 2000 (Invitrogen). *Mob1a/b* cDNAs were synthesized with total RNAs isolated from mouse IECs and cloned into a modified pcDNA3-Flag vector. These *Mob1a/b* expression plasmids were transfected in the Caco-2 cell lines using Lipofectamine 2000 (Thermo Fisher Scientific).

### Treatment of animals with the selective inhibitors

The γ-secretase inhibitor DBZ and Wnt pathway inhibitor Pyrvinum were purchased from Sigma-Aldrich, and BMP pathway inhibitor LDN193189 and TGF-β pathway inhibitor SB431542 purchased from ApexBio, Houston, TX, USA. All inhibitors were dissolved in DMSO (Sigma) and were suspended finely in 0.5% (w/v) hydroxypropylmethylcellulose and 0.1% (w/v) Tween 80 (Sigma) in distilled water. The final concentration of DMSO was 5%. The dosage of intraperitoneal administration of DBZ, Pyrvinum, LDN193189, and SB431542 were 3 μmol/kg, 5 mg/kg, 3 mg/kg, and 10 mg/kg, respectively. Intraperitoneal administration was performed for five days at intervals of 12 h after tamoxifen treatment.

### β-galactosidase staining assay

The intestines fixed on 3 mm paper were incubated in 2% PFA at 4 °C for 1 h and then rinsed with PBS 3 times (10 min each). The tissues were incubated β-galactosidase staining solution (1 mg/ml X-gal, 1 mM K_4_Fe(CN)_6_, 1 mM K_3_Fe(CN)_6_, 2 mM MgCl_2_, 0.005% deoxycholate and 0.01% NP-40 in PBS) at 4 °C for overnight in a dark condition. The stained tissues were rinsed with PBS three times and images were obtained using Stemi-2000c (Zeiss).

### Gene expression analysis by qRT-PCR

Total mRNA was isolated from intestinal epithelial cells using TRIzol^®^ reagent (Thermo Fisher Scientific). cDNA was synthesized with ReverTra Ace^®^ qPCR RT Master Mix (TOYOBO, Osaka, Japan). qRT-PCR was performed using LightCycler^®^ 480 (Roche, Basel, Switzerland) and 2× SYBR green premix (Enzynomics, Daejeon, Republic of Korea). Sequences of oligonucleotides are listed in Tables S4 and S5.

### Statistical analysis

Statistical analysis (unpaired two-tailed Student’s *t* test) were performed using the GraphPad Prism 5 software, CA, USA. *P* values of ^*^P < 0.05, ^**^P < 0.01, ^***^P < 0.001 were considered as significant with 95% confidence interval.

## Electronic supplementary material

Supplementary table 1

Supplementary table 2

Supplementary table 3

Supplementary table 4

Supplementary table 5

Supplemental figures

Supplementary information legends
