# Supplementary material for: Depletion of MOB1A/B causes intestinal epithelial degeneration by suppressing Wnt activity and activating BMP/TGF-β signaling
Source: Cell Death Dis. 2018 Oct 22;9(11):1083. doi: 10.1038/s41419-018-1138-0 (PMC6197243; doi:10.1038/s41419-018-1138-0)

**Supplemental Figure S1.**

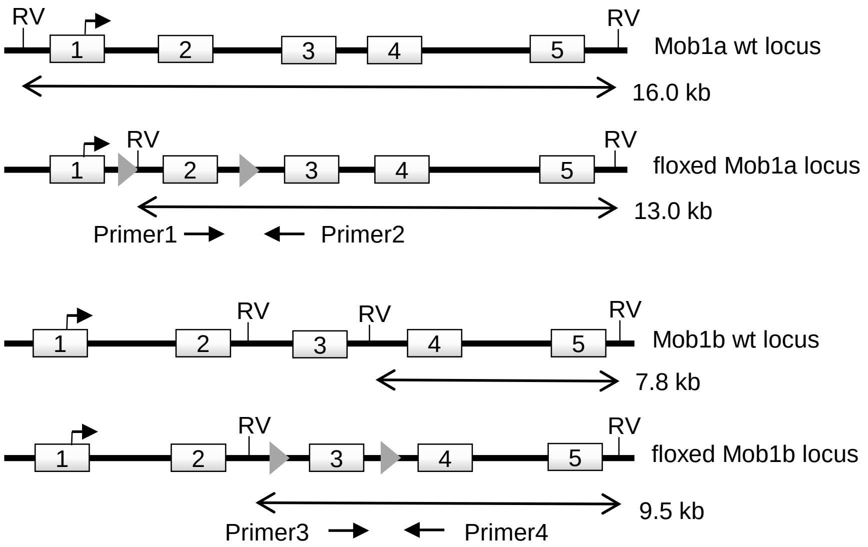


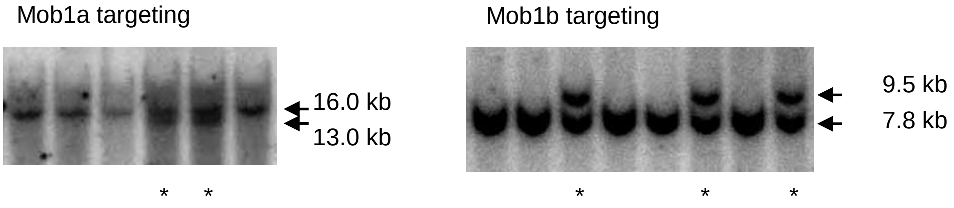


| PCR primer | sequence |
|---|---|
| Primer1 | CCTCGTCGTCTGGATCTAGC |
| Primer2 | CCAGCATACTGAAGACCACTC |
| Primer3 | CCATCTCTTCAGCCTCCCTTCTTG |
| Primer4 | GAGCTTAGTGCTAGAGAGATGAC |

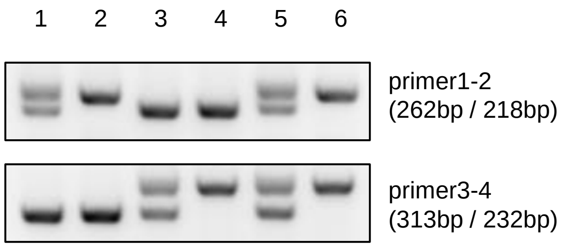

**Supplemental Figure S2.**

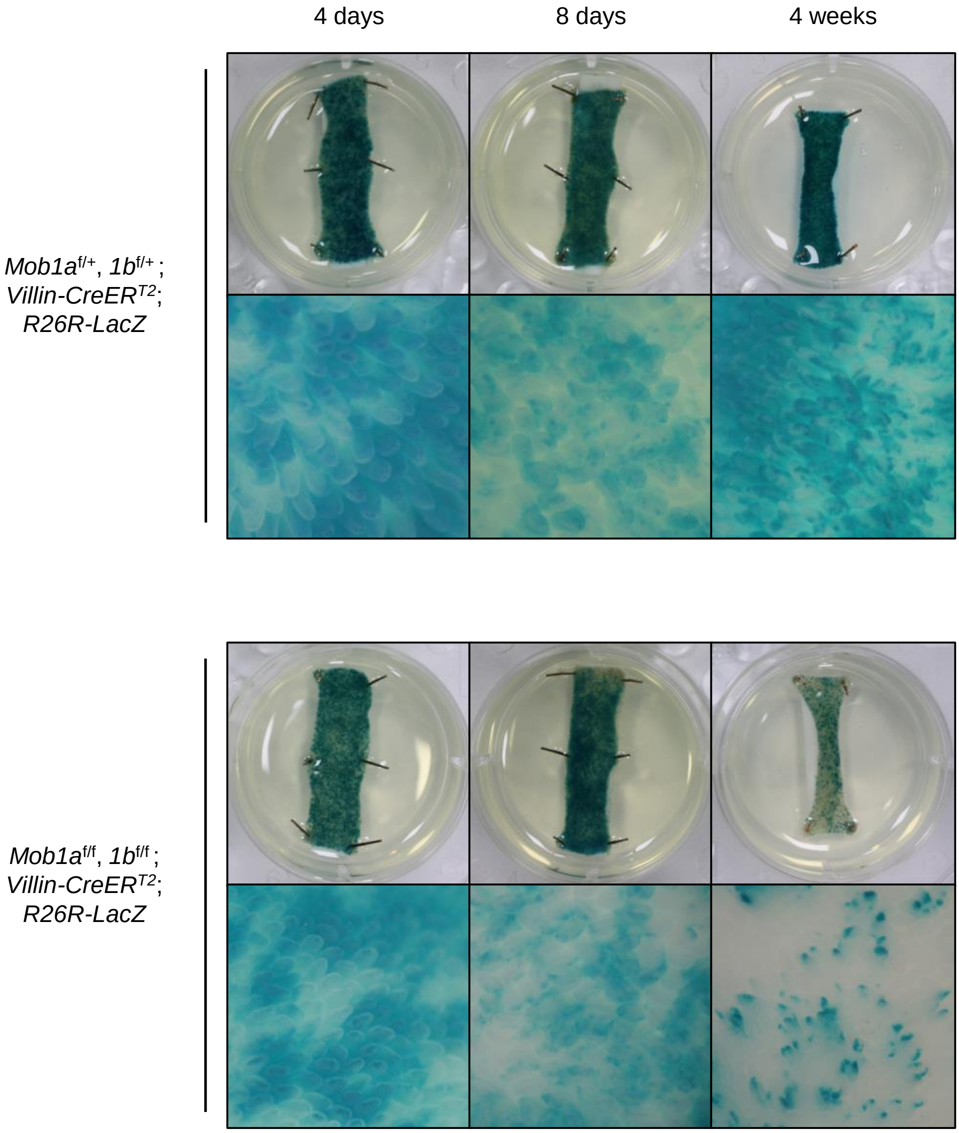

**Supplemental Figure S3.**

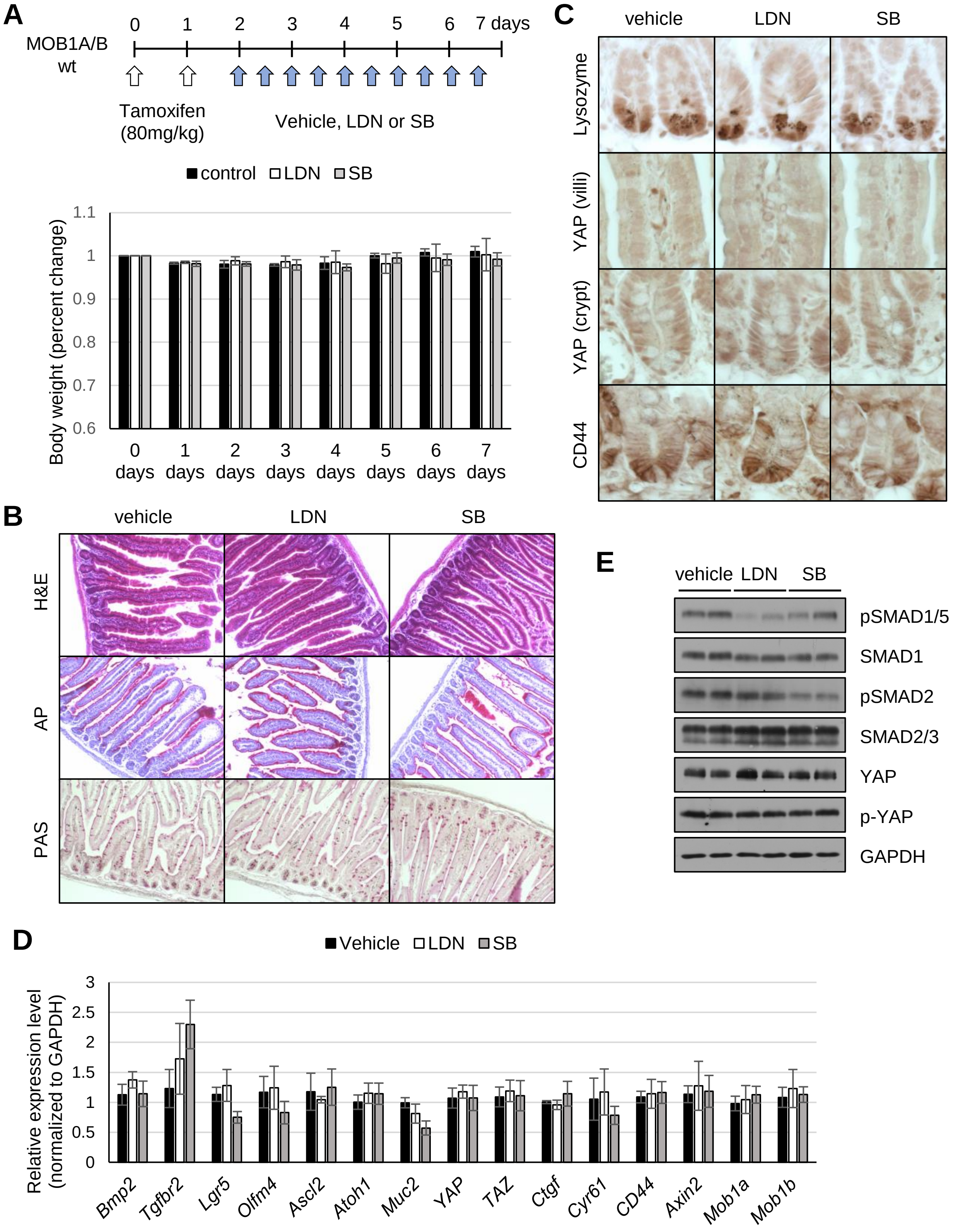

## Supplemental Figure S4.

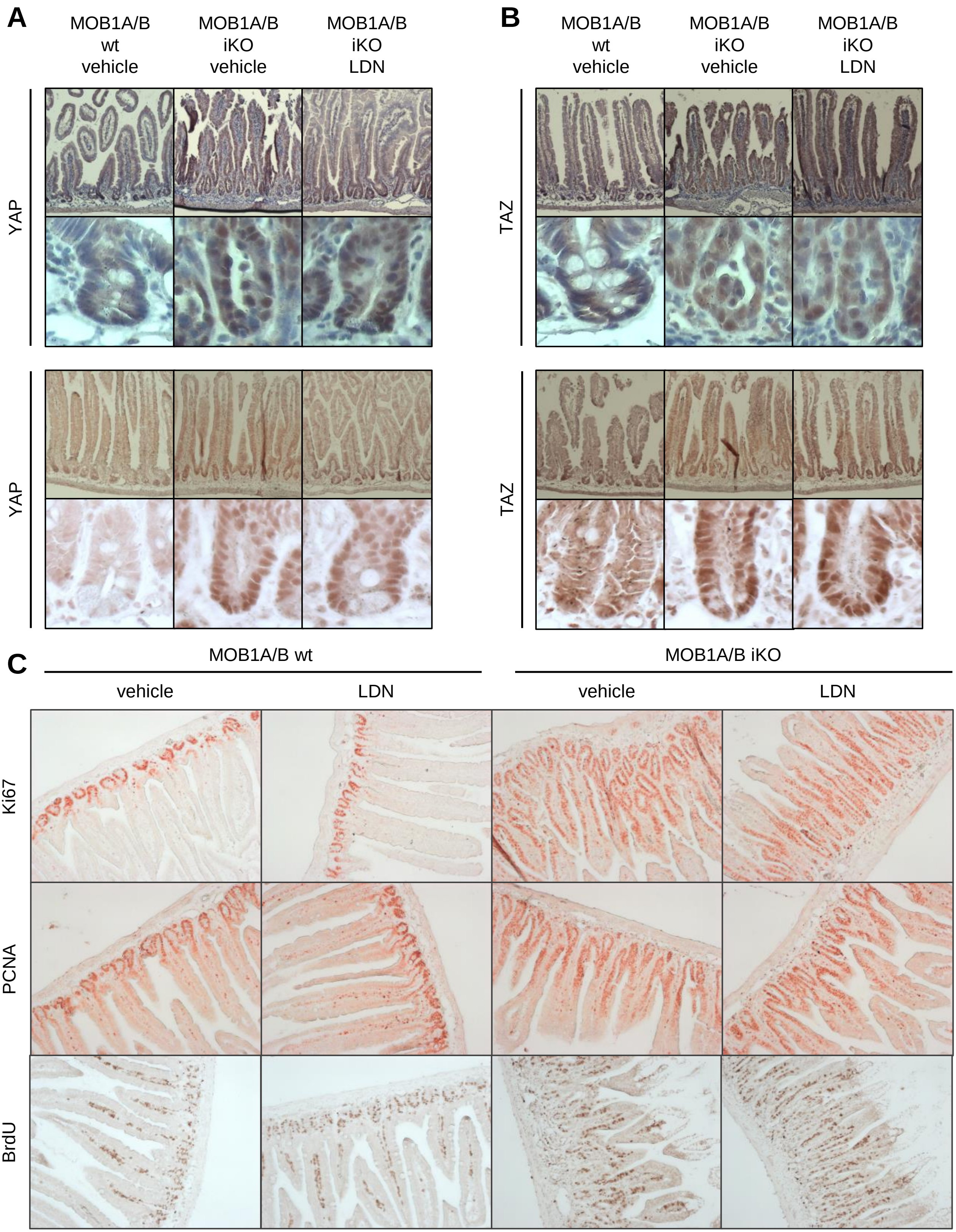

**Supplemental Figure S5.**

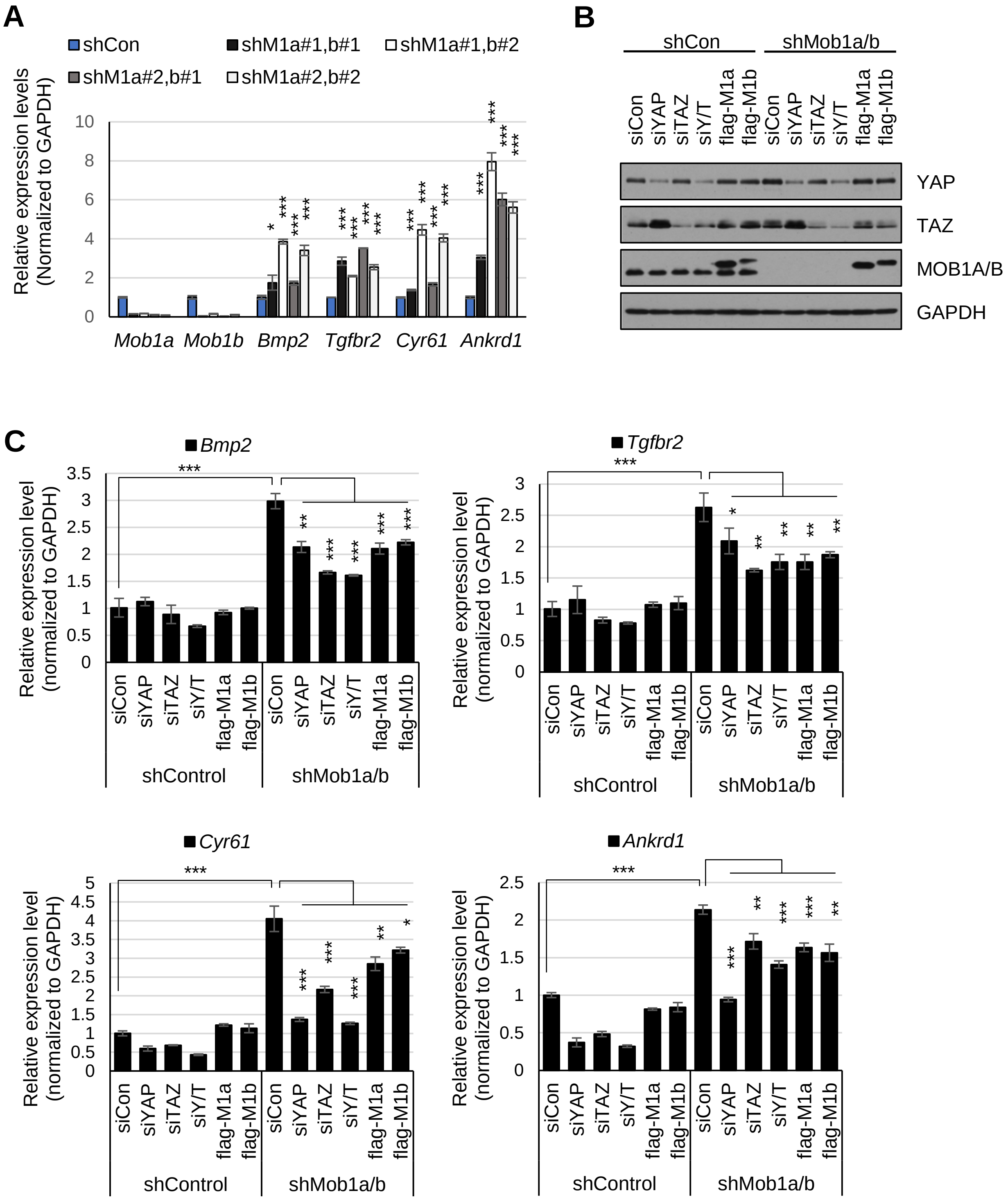

**Supplemental Figure S6.**

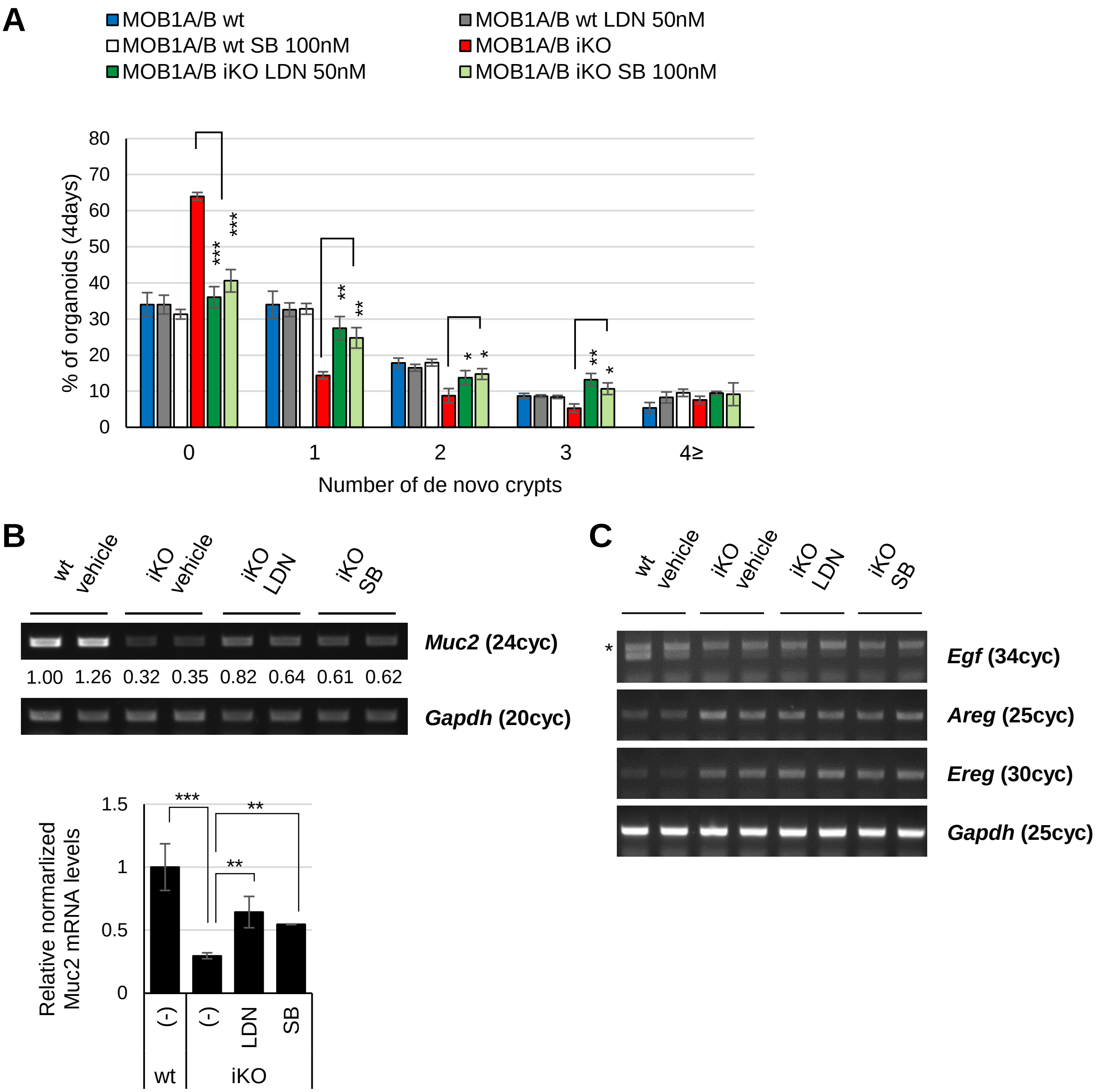

Supplement: Supplementary file 6 — Supplemental figures [file 41419_2018_1138_MOESM6_ESM.pdf]
