# Supplementary material for: Depletion of MOB1A/B causes intestinal epithelial degeneration by suppressing Wnt activity and activating BMP/TGF-β signaling
Source: Cell Death Dis. 2018 Oct 22;9(11):1083. doi: 10.1038/s41419-018-1138-0 (PMC6197243; doi:10.1038/s41419-018-1138-0)
Supplement: Supplementary file 7 — Supplementary information legends [file 41419_2018_1138_MOESM7_ESM.docx]

**Supplementary information legends**

**Figure S1. Schematic diagram of the generation of *Mob1a/b* gene-modified mice and genotyping.**

**Figure S2. MOB1A/B-depleted ISCs were negatively selected.** Bright-field images of whole mount β-galactosidase staining sampled on the indicated days after tamoxifen treatment (40mg/kg). The lower images are an enlarged view of the upper images.

**Figure S3. The effect of LDN- or SB treatment in MOB1A/B wt mice.** **a** Schematic diagram of tamoxifen and inhibitor treatment. Percent changes in body weight of vehicle- or inhibitor-treated MOB1A/B wt mice (n = 3) after tamoxifen treatment. **b, c** Representative hematoxylin and eosin (H&E) staining, alkaline phosphatase (AP) staining, periodic acid-schiff (PAS) staining and IHC staining with indicated antibodies of intestinal sample in (a). **d** Quantitative results showing expression levels of indicated mRNA from IECs isolated from vehicle- or inhibitor-treated MOB1A/B wt mice (n = 3). **e** Western blot analysis of IECs isolated vehicle- or inhibitor-treated MOB1A/B wt mice. Data are presented as the mean ± SEM.

**Figure S4. Localization of YAP and TAZ in LDN treated MOB1A/B iKO mice. a, b** Representative IHC staining results obtained with anti-YAP (a) and anti-TAZ (b) antibodies in intestinal sample isolated from vehicle- or inhibitor-treated three individual MOB1A/B wt and MOB1A/B iKO mice at 7 days after tamoxifen treatment. Upper images were counterstained with hematoxylin and lower images were not. **c** Representative IHC staining results obtained with anti-Ki67 (upper), anti-PCNA (middle) and anti-BrdU (lower) antibodies in intestinal sample isolated from vehicle- or inhibitor-treated three individual MOB1A/B wt and MOB1A/B iKO mice at 7 days after tamoxifen treatment.

**Figure S5. Upregulation of *Bmp2* and *Tgfbr2* by MOB1A/B knockdown was dependent on YAP and TAZ activities in Caco-2 cell line. a** Quantitative result showing expression levels of indicated mRNA from control or shMOB1A/B stably transfected Caco-2 cells. **b, c** Control and shMOB1A#2, MOB1B#2 Caco-2 cell lines were transfected with indicated siRNA or Flag-mouse MOB1A/B. Western blot analysis (b) and qRT-PCR (c) were performed at 48h after transfection. Data were collected from at least three independent experiments. Data are presented as the mean ± SEM. **P* < 0.05, ***P* < 0.01, ****P* < 0.001.

**Figure S6. Inhibition of BMP/TGF-β signaling recover the intestinal defects caused by depleting MOB1A/B. a** Percentage of organoids showing 0, 1, 2, 3 or ≥4 de novo crypts after 4-day culture of isolated ISCs. Culture media with indicated inhibitors were replaced every day. Results were obtained from three independent cultures. **b, c** Semi-quantitative PCR analysis of mRNA isolated from vehicle- or inhibitor-treated three individual MOB1A/B wt and MOB1A/B iKO mice at 7 days after tamoxifen treatment. Lower graph represent quantification of *Muc2* mRNA in (b). Upper band (*) of PCR amplification results using Egf primer was non-specific in (c). Data are presented as the mean ± SEM. ***P* < 0.01, ****P* < 0.001.

**Supplementary Table 1. MOB1A/B is essential for normal development of IECs during embryogenesis.** Viability of pups of the indicated *Mob1a/b* genotypes from crossbreeding with *Mob1a*^f/+^; *Mob1b*^-/-^; *Villin-Cre* and *Mob1a*^f/f^; *Mob1b*^-/-^ mice.

**Supplementary Table 2. Primary antibodies for immunohistochemistry.**

**Supplementary Table 3. Primary antibodies for immunoblotting.**

**Supplementary Table 4. Mouse primer sequence used for quantitative or semi-quantitative PCR.**

**Supplementary Table 5. Human primer sequence used for quantitative or semi-quantitative PCR.**
